# Insulin Resistance: Regression and Clustering

**DOI:** 10.1371/journal.pone.0094129

**Published:** 2014-06-02

**Authors:** Sangho Yoon, Themistocles L. Assimes, Thomas Quertermous, Chin-Fu Hsiao, Lee-Ming Chuang, Chii-Min Hwu, Bala Rajaratnam, Richard A. Olshen

**Affiliations:** 1 Google Inc., Mountain View, California, United States of America; 2 Division of Cardiovascular Medicine, Department of Medicine, Falk Cardiovascular Research Center, Stanford, California, United States of America; 3 Division of Biostatistics and Bioinformatics, Institute of Population Health Sciences, National Health Research Insititues, Miaoli County, Taiwan; 4 Graduate Institute of Clinical Medicine, National Taiwan University, Taipei, Taiwan; 5 School of Medicine, National Yang-Ming University, Taipei, Taiwan; 6 Department of Health Research and Policy, Stanford, California, United States of America; 7 Department of Electrical Engineering, Stanford, California, United States of America; 8 Department of Statistics, Stanford, California, United States of America; 9 Department of Environmental Earth System Sciences, Stanford, California, United States of America; Queen’s University Belfast, United Kingdom

## Abstract

In this paper we try to define insulin resistance (IR) precisely for a group of Chinese women. Our definition deliberately does not depend upon body mass index (BMI) or age, although in other studies, with particular random effects models quite different from models used here, BMI accounts for a large part of the variability in IR. We accomplish our goal through application of Gauss mixture vector quantization (GMVQ), a technique for clustering that was developed for application to lossy data compression. Defining data come from measurements that play major roles in medical practice. A precise statement of what the data are is in Section 1. Their family structures are described in detail. They concern levels of lipids and the results of an oral glucose tolerance test (OGTT). We apply GMVQ to residuals obtained from regressions of outcomes of an OGTT and lipids on functions of age and BMI that are inferred from the data. A bootstrap procedure developed for our family data supplemented by insights from other approaches leads us to believe that two clusters are appropriate for defining IR precisely. One cluster consists of women who are IR, and the other of women who seem not to be. Genes and other features are used to predict cluster membership. We argue that prediction with “main effects” is not satisfactory, but prediction that includes interactions may be.

## Introduction

An individual is considered insulin resistant if his or her insulin mediated glucose uptake by muscle and adipose tissue is impaired [1]. As a compensatory mechanism, beta cells in the pancreas secrete increased amounts of insulin in an attempt to maintain normoglycemia. This compensatory mechanism is termed hyperinsulemia. Because hyperinsulinemia has negative effects on tissues that are sensitive to insulin such as the liver and kidneys, it is not a benign condition.

Insulin resistance (IR) with compensatory hyperinsulinemia, increases the excretion of triglycerides by the liver, resulting in elevated serum levels of triglycerides; secondarily it results in decreased levels of high density lipoprotein cholesterol (HDL, the “good” cholesterol) as well as increased presence of small and more than normally dense low density lipoprotein (LDL, the “bad” cholesterol) particles. IR/compensatory hyperinsulinemia also appears to contribute to the complex pathogenesis of the most common form of elevated blood pressure, namely essential hypertension [2],[3] by promoting water resorption in the kidney and/or increased activity of the sympathetic nervous system. Through these and other “downstream” adverse metabolic consequences whose description is beyond the scope of this brief introduction IR/compensatory hyperinsulinemia markedly increases the risk of developing type 2 diabetes as well as various complications of atherosclerosis including coronary artery disease, ischemic stroke, and peripheral arterial disease even in the absence of diabetes.

Current conventional wisdom has it that an individual’s degree of insulin resistance can be estimated biologically most accurately and directly by one of two procedures. The euglycemic clamp is one; the insulin suppression test is the other. The two procedures produce measures of IR that are highly correlated, with a squared correlation coefficient 0.85 [4]. See also [5]. For purposes of research we have utilized the results of “steady state plasma glucose, ” or SSPG [5], the second procedure, for some subjects; comparison of our methodology for clustering and SSPG is reported in material that follows. Studies by which IR is estimated using such “gold standard” measures confirm a very wide range of insulin sensitivity in healthy, non-diabetic, non-hypertensive individuals. However, estimating IR using either of the most accurate procedures is invasive and laborious. Neither has made its way to the clinic thus far, and neither is likely to in the foreseeable future. As a consequence, multiple approximations have been developed. Given the critical need to measure IR precisely, the correlations of surrogate measures defined so far with putative gold standard measures are only modest (.5 to .7; [6], [7]). These approximations involve systemic background levels of glucose and insulin, which thus are “nuisance parameters”. Indeed, SSPG is one measure of the body’s ability to respond to a fixed glucose challenge where only a known fixed level of exogenous insulin is available. Since IR is inferred largely from higher-than-expected values of measured quantities such as glucose and insulin following a glucose challenge, and since “errors of measurement” are well known to attenuate “real” effects [8], [9], one might expect that IR as defined by surrogate measures underestimates actual incidence of IR by individual. This is borne out for those of our subjects who underwent SSPG.

Surrogate markers of IR include more easily obtainable measures of serum insulin and glucose over the two hour window of an oral glucose tolerance test (OGTT). As is done typically in medical practice, we also include measurements of serum lipids, including triglycerides, total cholesterol, LDL and HDL cholesterol. Heretofore, results of an OGTT and lipids have been taken to define IR, albeit subjectively. These informal definitions involve high triglyceride to HDL ratio, a homeostatic model assessment of insulin resistance (termed HOMA IR; see [10] for example), and high values of insulin “area under the curve” (AUC) for a period following a glucose challenge (for example, see [11]).

Conventional wisdom has it that two powerful determinants of IR are adiposity as estimated by body mass index (BMI) or waist circumference, and physical fitness best quantified by maximal oxygen uptake with exercise (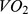 max). By conventional analyses, the two may explain up to 50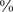 of the variation of gold standard measures of insulin sensitivity in linear models [12], [13], [14]. By determinants we mean both markers of those individuals who will develop IR and also of those individuals who already are IR.

## Methods

### Ethics Statement

The U.S. National Heart, Lung and Blood Institute’s (NHLBI’s) Family Blood Pressure Program (FBPP) was a large, genetic study of high blood pressure and related conditions in multiple ethnic groups. The program consisted of four networks: GenNet, GENOA, HyperGen, and SAPPHIRe [15]. SAPPHIRe stands for Stanford Asian Pacific Proram in Hypertension and Insulin Resistance. Each was funded by the NHLBI beginning in 1995. FBPP focus has been to identify genes that contribute to essential hypertension or related phenotypes through studies by linkage and (somewhat later by) association. For SAPPHIRe, IR was chosen as intermediate phenotype, by which we mean that abnormalities in blood pressure predispose to abnormalities in IR. We have results on only one OGTT per individual. Thus, data were gathered at the outset of an individual’s entry. Throughout this document, by *sibs* are meant sisters (or brothers) descended from the same set of parents. A *sibship* is a set of sibs. The individual by whom we identified sibships, that is, the proband was determined among Chinese in San Francisco, Hawaii, or Taiwan. IRBs (meaning Institutional Review Boards) of all the institutions of our collaborative study approved the research reported here. An individual was considered Chinese if all four grandparents self-reported as Chinese.

### SAPPHIRe Data Set

**Figure 1:**
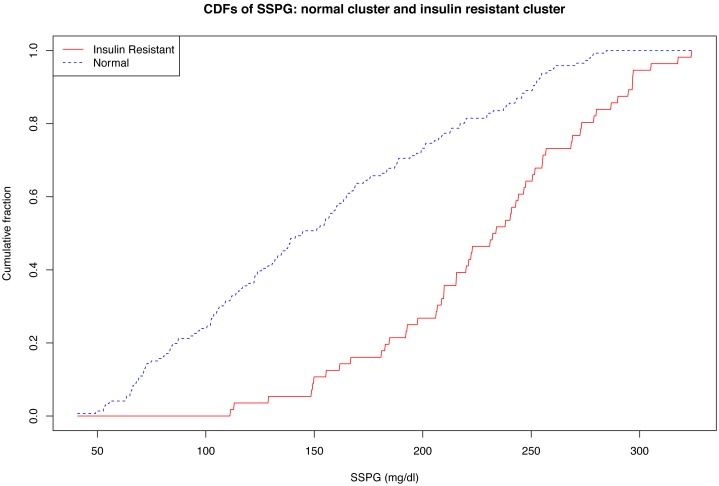
Clusters and SSPG. CDFs of SSPG: “Normal” cluster vs. “Insulin resistant” cluster.

SAPPHIRe recruited Chinese and Japanese hypertensive patients in two phases. The first, from which data here were derived, were from four hospitals in Taiwan, one in Hawaii, and one in the San Francisco Bay Area. In this first phase, a total of 1460 Chinese siblings (602 males and 858 females) from 557 families were enrolled. Through an interview, a physical examination, a blood draw, and an OGTT, 11 measurements (listed in Table 1) relevant to IR were obtained on all subjects. DNA was purified from whole blood and was used to genotype 293 SNPs in candidate genes. From the original sample we excluded 517 people who were missing at least one crucial measurement. After exclusion there were 943 sibs, 386 males and 557 females for whom results are presented here. SSPG was also obtained for a subset of 202 female participants. Results on SSPG as it corroborates (or not) clusters computed in what follows are reported in Figure 1. Each sibship studied contained one proband for hypertension, that is, a subject who presented as hypertensive Sibships involved hypertensive and other (typically hypotensive) sibs of the proband. SAPPHIRe data analyzed here were not gathered from a population study. This concern for conditional rather than unconditional attained significance and related applies to many epidemiologic studies.

**Table 1:** Eleven measurements relevant to insulin resistance.

Variables	Description
Age	age at exam, years
BMI	body mass index, kg/m^2^
Triglyc	Triglycerides, mg/dl
TCHL	total cholesterol, mg/dl
HDL	HDL cholesterol, mg/dl
OGTTG0	oral glucose tolerance testing of glucose at baseline, mg/dl
OGTTG1	oral glucose tolerance testing of glucose after one hour, mg/dl
OGTTG2	oral glucose tolerance testing of glucose after two hours, mg/dl
OGTTI0	oral glucose tolerance testing of insulin at baseline, mg/dl
OGTTI1	oral glucose tolerance testing of insulin after one hour, mg/dl
OGTTI2	oral glucose tolerance testing of insulin after two hours, mg/dl

We had 293 single nucleotide polymorphisms (SNPs) genotyped in a total of 57 “candidate genes,” the respective proteins for which they code bearing upon hypertension. These SNPs are listed in [16]. In particular, many such genes were chosen by resident SAPPHIRe experts because their proteins influence blood pressure control or glucose homeostasis. For example, APOAV regulates triglyceride levels, which are known to be differentially expressed in insulin resistance [17]. CD36 is a multifunctional receptor [18] that plays a part in mediating intracellular signaling as well as in taking up biologically active substances such as long-chain fatty acids. Primers were designed to sequence the promoter region, the 5′ and 3′ untranslated regions (UTRs), the exons, and the intron-exon boundaries of each candidate gene. A discovery set comprised of 24 SAPPHIRe individuals’ DNA was sequenced to identify SNPs and intertion-deletion mutations. These individuals were chosen to be hypertension, though implicitly we used information also on normotensive individuals because we know the “wild type” value at each SNP. After assembling the sequence contigs using the program Consed [19], the SNPs were tagged [20] and called manually in each of the 24 individuals. The SNPs identified in this manner were cross-checked against the public dbSNP database [21] and entered into a hand-curated SNP report. In general, SNPs not in high linkage disequilibrium (LD) with each other, that have greater than approximately a 10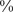 allele frequency (see Table 2), and that were deemed likely to change protein function were chosen for genotyping. We believe that including these 24 individuals in our analysis has introduced approximately no bias in any conclusions. Genotyping was performed using the ABI Taqman 5′ nuclease allelic discrimination system with either custom made or commercially available primers and probes. The accuracy of the genotypes was tested by comparing them against the discovery set sequences and against a 15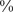 repeated set of DNA.

**Table 2:** Details of SNPs found to be predictive of insulin resistance.

SAPPHIRe terminologyfor predictiveSNPs	dbSNPAccession	Humangene	Location	Frequency ofmajor allele	Mutualinformation
LAMA4_S.2	rs1050348	LAMA4	6q21	0.82	0.0177
CYP1B15		CYP1B1	2p21	0.82	0.0153
LAMA4_S.17	rs1050353	LAMA4	6q21	0.66	0.0170
LAMA4_S.22	rs12208872	LAMA4	6q21	0.66	0.0162
LAMA4_S.18	rs3734289	LAMA4	6q21	0.66	0.0157
FOXO1A_S.4	rs3751437	FOX01	13q14.q	0.91	0.0148
APOAV_S.6	rs662799	APOAV	11q23	0.74	0.0141
APOAV_S.1	rs2072560	APOAV	11q23	0.74	0.0135
SLC2A4_S.1	rs5435	SLC2A4	17q13	0.7	0.0136
HUT2SNP5	rs1123617	HUT2	16q21	0.68	0.0084
PRKCI.2	rs55683301	PRKC1	3q26.3	0.93	0.0104
CD36.1	rs1405747	CD36	7q11.2	0.5	0.0107
CD36.3	rs3211956	CD36	7q1.2	0.75	0.0106

The genetic data used in our study were generated before the current era of high-density arrays, and before the U.S. NIH policy requiring that genetic data paid for with NIH monies be shared publicly through dbGAP. Indeed, sharing data is not legal even if the data are de-identified because the subjects did not consent to such sharing. We acknowledge that most IRBs have been willing to allow such data sharing without re-consenting so long as access is controlled. Thus, data will be made available to any qualified investigator who wishes to work with them. He or she should contact the senior author, Richard Olshen (olshen@stanford.edu) with a proposal for a manuscript. Olshen will survey SAPPHIRe investigators who remain alive (several are coauthors of this paper), and will reply to any proposer.

### Why we Restrict this Analysis to Chinese Women

The Chinese population is about 19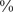 hypertensive (see [22]). Generally speaking, hypertension in the Chinese does not owe to their being obese (see [23]). Given the greatly narrower range of BMI in Chinese than in other populations, it is plausible that there is less variability in BP in Chinese than in other populations (see [23]). Further, since men tend to be more hypertensive than women (see [24]), we began by thinking that the prevalence of hypertension, which is highly correlated with IR in men, owes more in men to obesity than it does in women. Remember that IR was chosen as an intermediate phenotype by SAPPHIRe investigators. Therefore, if one looks for genes that predispose to either hypertension or IR, it seems reasonable to study Chinese women, as we have done. And remember, too, that the FBPP was given as its initial task to hunt for single SNPs in which “abnormalities” predispose to hypertension. By abnormalities we mean mutation away from the “wild type,” the prevalent genotype.

### How we Pick Regressors and why we Regress out Age and BMI

**Figure 2:**
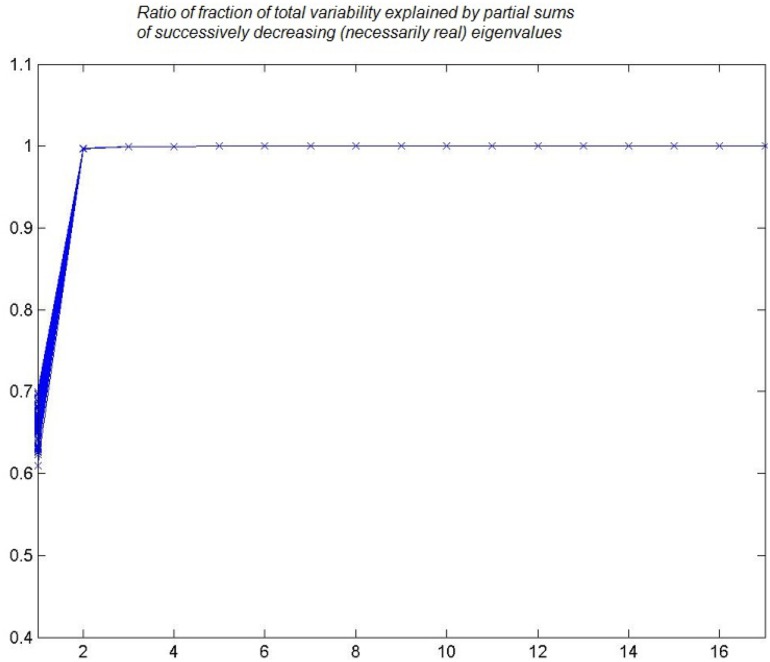
Scree plot. Eigenvalue ratio of 1,000 bootstrapped samples.

Conventional wisdom has it that age and BMI both influence IR and hypertension [25],[26]. Because our interest is in genes, abnormalities in which predispose to IR, it seemed particularly important to us to remove the impact of age and BMI on IR. Therefore, we employed linear regression to remove the impact of age and BMI on nine key variables by which IR is quantified. They are triglycerides; total cholesterol; HDL; and measured glucose and insulin at baseline, one hour, and two hours as part of an OGTT. We began our search for regressors by studying 17 simple functions of age and BMI that owe to Maclaurin and Fourier expansions of these two variables and functions of them. The 17 are AGE, BMI, 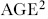, 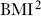, 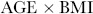, 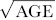, sqrt(BMI), log(AGE), log(BMI) sin(AGE), sin(BMI), cos(AGE), cos(BMI), sin(AGE/2), cos(BMI/2). Each individual thus defines a point in 17-dimensional Euclidean space. One can compute principal components (eigenvectors) of these 17-dimensional vectors to see what summarizes their variability. Indeed, one can infer the percent of mean-square variability explained by cumulative successive principal components from the “Scree plot.” Rather than simply compute the successive fractions, we instead bootstrapped individuals according to principles that are specific to our dataset and their inferred cumulative percent explained. Exactly how the bootstrapping was accomplished is described in what follows. But readers are referred now to our summaries in Figure 2.

A cursory examination of the principal components demonstrates that the variability of the 17-dimensional vectors is explained by the first two principal components, and that these first two principal components depend crucially on only three functions of AGE and BMI, namely 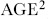, 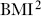, and the product 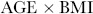. In order to avoid beginning our explanations with quadratic terms, we added in the two corresponding first order terms to come up with five terms, the cited three plus AGE and BMI themselves. Of course, one might have intuited this from Maclaurin expansions, but better in our view “to let the data speak for themselves”.

### How we Bootstrap our Data

The detailed form of bootstrapping in our scenario is rather tricky. We tried to be faithful to the bootstrap principle that sampling with replacement from the empirical distribution of the data bears approximately the same relationship to that empirical distribution as does the empirical distribution to “nature.” However, “The empirical distribution of what?” Most individual measurements are not independent of all others since the measurements are for sibships. We required sampling with replacement to be faithful to three principles. (1) The number of sibships in each bootstrap sample should be the same as the total number of sibships in the empirical distribution of our SAPPHIRe data (in particular, 287). (2) The expected number of people in each bootstrap sample should be the same as the total number of people in that empirical distribution. (3) For each 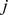, the expected fraction of sibships of size 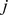, should be the same as in the empirical distribution. Readers will check easily that all three requirements are met if we sample sibships with replacement 287 times, but with the probability of sampling any particular sibships being proportional to its size. This is the same as sampling 287 individuals at random and with replacement, but then letting a bootstrap sample consist of individuals chosen and their respective sibships.

### How we Cluster Individuals

Clustering is applied twice to our data, one concerning certain residuals with units of clustering being individuals. That clustering is accomplished by methodology described in this subsection.

Vector quantization (VQ) design [27] as applied here amounts to a particular approach to clustering data. In VQ an input vector is represented by one of a predefined set of patterns (cluster centers = codewords). Data are assigned to cluster centers on the basis of which pattern is closest to the given input vector. VQ has been used successfully in pattern recognition, including speech and image processing [28],[29],[30]. VQ design can be viewed as fitting a model when partition cells are represented by their conditional probability density functions with respective weights given by estimated prior probabilities. VQ of dimension 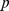 (i.e., the number of features is 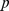) and size 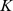 (the number of clusters is 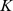) can be described by the mappings and sets: an *encoder* 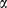, a *decoder* 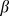, and a *length function* 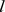. An *encoder* 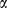 is a mapping of an input vector 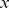 in 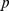-dimensional Euclidean space, 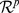, into an index 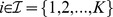. The encoder is described by a partition 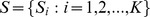 such that 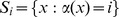. A *decoder* 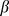 converts the index into a source reproduction 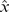, and 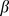 is associated with a reproduction codebook 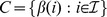. Finally, a *length function* 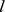 measures the complexity or cost of an index 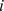, and it is “admissible” if 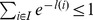. Both 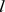 and the requirement of admissibility are closely seen to be related to the “prior probability” of the cluster indexed by 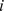. For a *fixed-rate* quantizer, 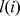 is fixed at [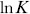] (the integer part of 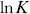) for all 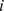. Otherwise, a quantizer is said to be *variable-rate*. Eq. (1) summarizes VQ.
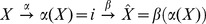
(1)


Here, for purposes of defining insulin resistance on the basis of certain residuals, we are interested in GMVQ, where we fit Gauss mixture models (GMM) to data in a VQ design using the Lloyd algorithm with a suitable distortion measure [31],[30]. The EM algorithm [32] is the most popular approach to fitting a GMM to data, but the Lloyd algorithm is one alternative. The main difference between the Lloyd and EM algorithms is that in most implementations EM entails soft decisions, whereas the Lloyd algorithm entails hard decisions. The EM fits a GMM to each observed vector, whereas the Lloyd fits a single component of a GMM to each observed vector. This “hard” assignment of components to observed data is based on the information theoretic property of a Gaussian being a “worst case” for designing robust compression/source coding systems [30],[33].

In GMVQ, each cluster is represented by its prior probability 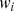 (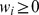 and 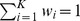) and a cluster conditional probability density function (pdf) 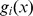, a multivariate Gaussian:
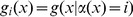
(2)

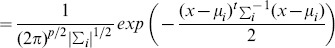
(3)where 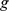 is a fitted GMM, and 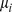 and 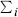 are the mean vector and covariance matrix of cluster 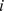, respectively; we assume 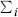 to be non-singular.

In GMVQ, we try to minimize the mismatch between the true pdf 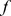 and the fitted model 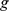 by iterating the Lloyd optimality conditions. The encoding rule (or cluster assignment rule) is to find a component 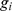 that minimizes the distortion 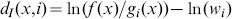. Since 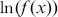 is common to all 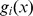, the encoding rule becomes:
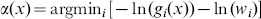


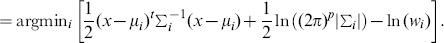



When the true pdf 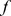 is a GMM, minimizing the distortion 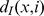 is equivalent to a maximum a posteriori selection (MAP) of a Gaussian model from a GMM (a collection of Gaussian models 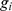 with a probability mass function 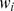) [30]. The MAP selection of 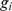 is
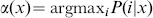


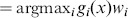


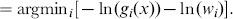



In GMVQ, we denote 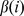 ( = centriod of 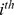 cluster) by 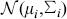, equivalently 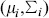.

The distortion measure between 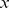 and 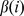 can be expressed:
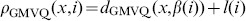
(4)where 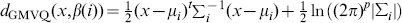, and 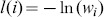. Finally, the average distortion in GMVQ becomes:
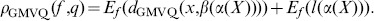
(5)When the underlying pdf 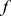 is unknown, typically the case in practice, the expectations in (5) become sample averages if an empirical distribution is used in the expectation:
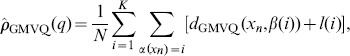
(6)where 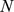 is the number of samples.

In GMVQ, the optimal 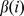 for a given encoder 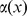 is defined by 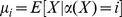 and by 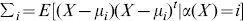. The optimal length function in GMVQ is 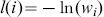, where 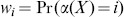. See [33], [30] for more details. In practice, the conditional expectations become conditional sample averages when we run the Lloyd clustering algorithm on a set of samples. To avoid singular covariance matrices, regularization as in [34] can be used.

GMVQ was used to cluster people. It performs well in many areas [35],[30],[36],[37],[29]. When GMVQ was applied to cluster people, the clustering was based on residuals from linear regression models described earlier. Insulin resistance was defined by clustering people, and the clustering model was validated internally. Therefore, we are trying to solve an unsupervised learning problem: we not only need to cluster people, but also we want to estimate the “true number” of clusters.

We tried first to estimate number of clusters by observing the GMVQ distortion in (6) as the number of clusters varied.

### A Permutation Test for Familiality

Recall that SAPPHIRe was initially a study by linkage of hypertension with IR, crudely defined, as the intermediate phenotype. Obviously, our study is of sibships, even though individuals were the sampling units. However, recruitment was not done in any sense that could be described as “random.” The proband was necessarily hypertensive. Over more than a decade SAPPHIRe policies for recruitment changed. They were influenced greatly by Risch and Zhang [38], although their paper concerned mapping quantitative trait loci (QTLs), not association. They recommended extremely discordant sib pairs in order to increase the power of studies when the probability of type 1 error is fixed. After 1995, the SAPPHIRe policy became an attempt to recruit hypotensive sibs of a hypertensive proband. Obviously, this reduced familiality of hypertension. Since hypertension and IR are related, adherence to this policy would also reduce familiality of IR. Adherence was particularly strong in Taiwan, from where a majority of SAPPPHIRe subjects were recruited. Nonetheless, it behooved us to investigate familiality of our clusters.

Denote the 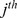 family by 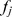 and the cardinality of its sibship by 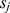. Apparently 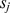 is an integer between 1 and 6, and 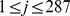. We denote people who are assigned to cluster 1 (our insulin resistant cluster) by 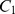 and those assigned to cluster 2 by 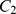. Familiality or lack of it is inferred by a conditional permutation test. For each cluster we compute the expected number of families that would appear in the cluster under a null hypothesis in which people are assigned to clusters at random without regard to sibship but three observed outcomes are fixed. When the number of families is denoted by 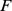, and the total number of individuals is denoted by 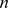, then 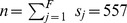. Count the number of families that appear in 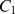:
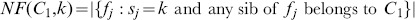


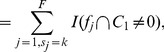
(7)where 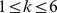.

Our test is conditional upon 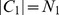; 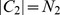; and 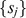. The null hypothesis, 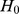 is that given the three cited conditions, people are assigned to clusters at random; and the (conditional) expected numbers of families of respective sizes 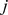 (2 through 6) are as if so assigned. One computes the conditional expectation:
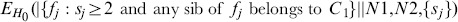


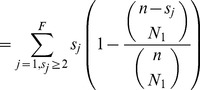
(8)


Table 3 gives sibship sizes (when it is at least two), total numbers of families, observed numbers of families of respective sizes, and numbers expected under 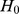. One needs no statistician to infer that by any reasonable test statistic, our null hypothesis is “accepted.” The third and fourth columns of Table 3 pass what the late Leonard J. Savage called “the traumatic intraocular test”.

**Table 3:** Sibship size and expected number of families in insulin resistant cluster under 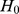.

Sibship size	num of families	*NF(C*_1_ *,k)*	expected num offamilies in *C*_1_
2	120	61	66.38
3	40	30	28.07
4	15	14	12.02
5	5	4	4.34
6	1	1	0.91
Total	181	110	111.72

### Inference from SNPs, Including Imputation

SAPPHIRe includes information from genotyping 293 SNPs in candidate genes that were selected because abnormalities in them are thought to predispose to insulin resistance. These data are *unphased* as to chromosome. Each SNP is coded as to the number of “major alleles,” that number being 0, 1, or 2. This section is about how we quantify the relationship of these SNP values and cluster membership. We summarize predictability by *mutual information* [39].

The *mutual information* between two (discrete) random vectors X and Y is a generalization of Kullback-Leibler distance and is defined as
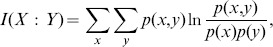
(9)where 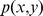 is a joint probability mass function (pmf) of 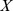 and 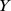, and 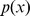 and 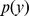 are marginal pmfs of 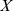 and 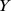, respectively. Obviously if 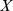 and 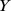 are independent, 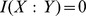. Mutual information has been used often in statistical learning [40]. Mutual information is always non-negative. The more 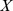 and 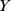 are related, the higher 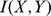 is. Thus, we postulate that SNPs relevant to insulin resistance have higher mutual information with insulin resistance than do other SNPs that are not. Once relevant SNPs are identified, we also try to find their interactions with environmental variables (age and BMI). Since insulin resistance can be triggered by environmental factors, SNPs alone do not cause all insulin resistance; and both (relevant) SNPs and their interactions with age and BMI are used to classify people based on our definition of insulin resistance. Five classification algorithms were considered to evaluate the procedure for finding relevant SNPs and their interactions with environmental variables. In using relevant SNPs to train classifiers, we tried to remove redundancies. Two SNPs are taken to be similar if they have high enough mutual information. SNPs were clustered so that clusters consist of groups of similar SNPs.

Among the 297 SNPs, 36 SNPs had constant value; so, obviously, they were useless for prediction. For 12 SNPs, fewer than 400 people were genotyped. We therefore discarded these 48 SNPs from subsequent analyses. For the remaining 249 SNPs, missing values were imputed by RPART [41]. We realize that there are other approaches to imputation, such as IMPUTE [42], [43], MACH [44], [45], and Beagle [46], and our use of RPART disregards such as published values of linkage disequilibrium and information from the HapMap project [47], [48]. For technical reasons, it was possible to try only MACH among the three cited programs. It did not give imputations that were better than our imputing method RPART for those sequences and sites for which we had a “gold standard.” Individuals with more than 110 missing SNP values were discarded prior to imputation. This reduced the number of subjects from 557 to 485.

### Clustering SNPs

Even after imputation, there were SNPs that were highly predictable from each other. These SNPs were combined by agglomerative clustering with average linkage [49],[40] employed to measure their similarity. Since each SNP can take three possible values (major allele homozygote, heterozygote, and minor allele homozygote), a 3
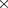
3 table was formed for each pair of SNPs, of which examples are given in Tables 4 and 5. For example, in SAPPHIRe terminology (see Table 2 for its translation to more customary dbSNP accession for all but one SNP) APOAV
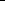
S.1 and APOAV
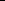
S.4 were almost perfectly predictable from each other as is clear from Table 4. Table 4 and Table 5 give examples in SAPPHIRe terminology for which the former exhibits high mutual information and the latter low mutual information. Table 2 summarizes the relevant SNPs that we found.

**Table 4:** 3
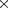
3 table of APOAV_S.1 vs. APOAV_S.4 (mutual information: 1.2675).

	BB	Bb	bb
AA	266	0	0
Aa	1	175	0
aa	0	0	37

**Table 5:** 3
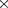
3 table of LEPR.12 vs PRKCZ.14 (mutual information: 0.0018).

	BB	Bb	bb
AA	94	35	0
Aa	59	32	0
aa	4	1	0

To perform agglomerative clustering, we grew a bottom-up-tree (dendogram) using (9) as a measure of similarity and continued to merge clusters until we were left with one. Since SNPs belonging to the same node are similar, we represented each node of the tree/dendogram (including terminal/leaf nodes) by a SNP with maximum mutual information with cluster membership among all SNPs belonging to the same node. We cut the tree; the SNPs representing terminal nodes of the resulting sub-tree became the final selection of relevant SNPs. Cross-validation was employed to decide where to cut the tree, accuracy of classification of cluster membership being the criterion.

### Predicting Cluster Membership from SNPs and Finding Interactions among Predictors

To evaluate the entire [5] procedure of predicting cluster membership from SNPs, AGE, 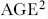, BMI, 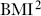, and 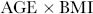 and (separately) interactions of them, we employed five state-of-the-art algorithms for prediction and did 10-fold cross validation of the entire process. Our choice was governed by previous successful application of the algorithms to genetic problems. Those we chose were support vector machines (SVM) [50],[51], the L1 (Lasso) regularization path algorithm for generalized linear models [52], logistic regression with L2 penalty [53], FlexTree [54], and random forests [6].

We have emphasized that IR can be triggered by genes, environmental features, and their interactions. Our discovery of interactions among predictors is by an approach that has proven useful in other contexts but that may be reasonably new to genetics [55]. Features for us were SNPs selected as described and the three scaled functions of age and BMI (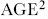, 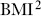, 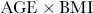) that, as we report in what follows, contain the great preponderance of information in the first two principal components of the 17 predictors that have been cited. The technology is CART, Classification and Regression Trees [56]. Because CART does not change with (that is, is “equivariant” to) monotone transformations of coordinate axes, the squares could have been deleted, and are in results we report. The “outcome” for our “two-class problem” is cluster membership, where clustering was performed as we have described. We anticipate results reported in what follows by reporting here that there was remarkable evidence for two clusters in our data. For our implementation of CART, we took the product of empirical frequencies of cluster times loss for a mistake to be equal by cluster. This choice is in keeping with the findings of [57]. Our CART decision tree was grown using RPART [41], the open source version of CART.

Precise methodology for selecting interactions depends on each path from root node of our rooted, binary decision tree to terminal nodes of the tree. If, for example, an optimally chosen [6] path is, “split on A, split on B, split on A,” then there are three “words” suggested: A, B, A 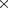 B if adjacent nodes are taken as suggestive of a two-factor interaction, and three- and higher factor interactions are ignored. This, then, is the basis for the three main effects and interaction chosen for inclusion in subsequent classifiers, which promise to be more accurate than nave CART itself and for which results are given in Discussion. Our approach harks back to the origins of binary tree-structured decision trees as, “automatic interaction detectors,” [58], and is particularly important in the typical polygenic context where there are so many candidates for features available, especially interactions. Note that with any word selected for inclusion, necessarily all “sub-words” are also included.

## Results

### Comparison of OGTT and SSPG for those Subjects for Whom we had both

There was no particular relationship between those subjects for whom we had SSPG as well as OGTT, and those for whom we did not. Given this haphazard selection, it is interesting to note from Figure 1. the relationship between the two. Obviously, were SSPG ever the standard in clinical medicine, this would be a different paper.

### The Scree Plot and Principal Components

We employed the time-honored technique of “principal components” [59] to decompose the variability in the 17 functions of AGE and BMI into their variability. Of course, we expected that far fewer than 17 dimensions would be required, and the Scree plot [60] that we report in Figure 2 indicates that our intuition was correct. The plot summarizes fraction of variability explained by respective “orthogonal” linear combinations of the 17 that summarize their variability. We were careful to bootstrap the principal components by the approach we invented for this purpose and that is reported in Section 4. Readers please note from the figure that only two principal components summarize approximately all the variability in the 17. Further, from computer output not repeated here it was altogether evident that these first two principal components could be computed with high accuracy from only the three functions that were cited in the previous section, namely, 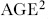, 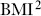, and 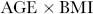.

### Numbers of Clusters

**Figure 3:**
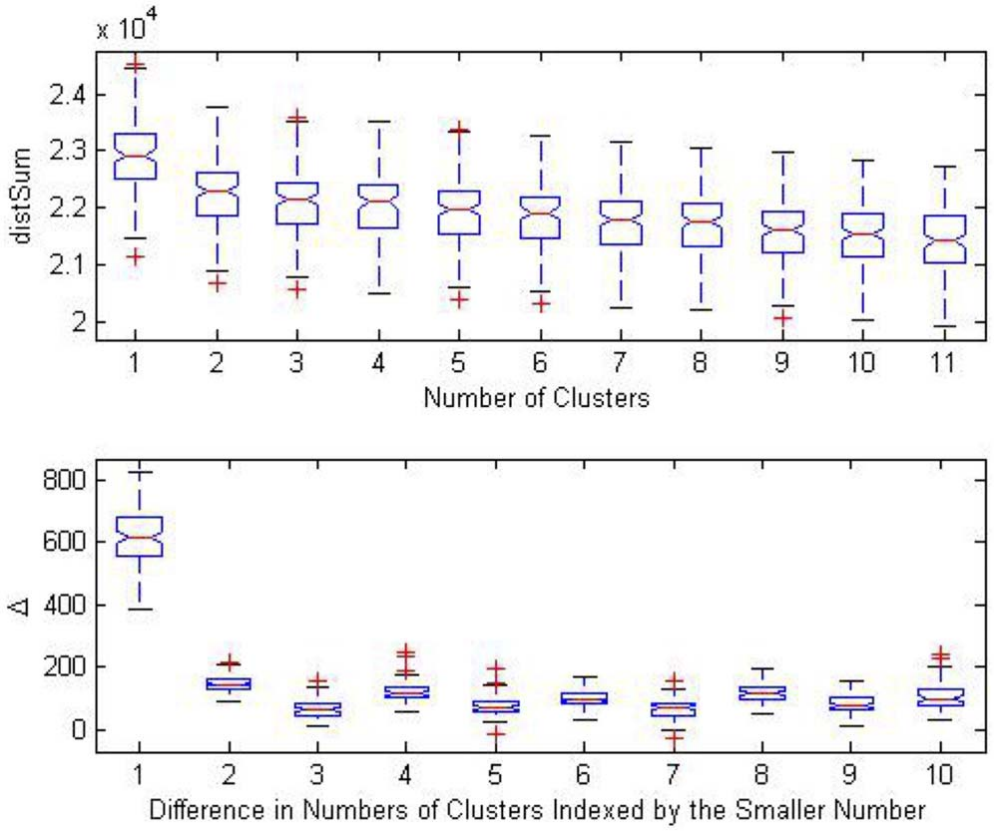
Estimation of number of clusters. Top: 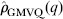 vs. number of clusters of 1,000 bootstrapped samples. Bottom: 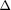 (decrease in 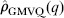) vs. difference in numbers of clusters indexed by the smaller number; 1,000 bootstrapped samples.

Clustering was performed with TSVQ as cited, with results summarized in Figure 3. Individuals were the units by which we clustered; what was actually clustered were residuals by subject of nine clinical features with the cited functions of AGE and BMI regressed out. Note from Table 1 that the clinical features were the six values of glucose and insulin measured by OGTT, triglycerides, total cholesterol, and HDL. Readers will note the two graphics, one giving a number proportional to the average “distortion” between a sample point and its “cluster center” as the number of clusters grows. Necessarily this number decreases with number of clusters. The second part of the figure gives the difference in distortion between successive numbers of clusters as their number grows. It is patent from the second graphic, but even the first, that our data suggest two clusters. We note that both the GAP statistic [61] and the silhouette method [62] were applied in order to estimate the “correct” number of clusters; both give strong evidence that two is the right number.

### Differences between Clusters

While clustering was performed on residuals, when we summarize cluster membership in Table 6 we added in fitted values so that means and standard deviations are for the original clinical measures by subject, by cluster. Of course, we include AGE and BMI themselves for a full summary. Were we successful in defining clusters that exist in our population of Chinese women, then AGE and BMI should not differ by cluster. However, the other nine features “should.” While marginal sampling distributions of the clinical features preclude any one statistical test being just right for comparing clusters, and we are certainly are sensitive to the matter that the features themselves were used to define the clusters, rendering attained significance of differences between clusters suspicious at best, we did nonetheless undertake comparison by an approximate Behrens-Fisher-Welch t-statistic, a permutation pooled t-statistic, and a Mann-Whitney statistic. Generally speaking, differences of lipids (by which we mean features that explicitly involve triglycerides or cholesterol) by cluster tend to be somewhat less than differences obtained from the OGTT. Comparison by pooled t might be considered valid if it is taken to be unconditional with respect to an unspecified mechanism that generated equal intrinsic variability per feature per subject.

**Table 6:** Cluster statistics based on B-F-W tests.

Medicalmeasurements	Cluster1*:Mean (STD)	Cluster2*:Mean (STD)	Behrens-Fisher-Welch t-statistic
Age	50.60 (8.57)	48.94 (8.50)	2.13
BMI	25.74 (3.51)	24.14 (3.45)	5.04
Triglycerides	55.66 (97.61)	92.28 (38.01)	8.35
Total cholestrol	198.82 (45.38)	185.33 (36.85)	3.46
HDL	43.47 (10.36)	49.81 (12.64)	-6.26
OGTT glucose t = 0	97.16 (15.27)	87.18 (9.38)	8.02
OGTT glucose t = 60	204.17 (44.29)	157.69 (39.04)	11.96
OGTT glucose t = 120	167.02 (55.23)	131.59 (32.45)	7.92
OGTT insulin t = 0	10.79 (6.29)	5.86 (2.93)	9.94
OGTT insulin t = 60	121.35 (81.75)	53.18 (24.88)	10.86
OGTT insulin t = 120	110.54 (78.37)	49.30 (30.26)	10.05

*Cluster1 and Cluster2 have 177 and 380 individuals, respectively.

No matter the caveats of the previous paragraph, we did attempt to quantify differences in the two clusters. Testing is conditional on there being 177 individuals in one cluster and 380 in the other. By “permutations” is meant the random division of the 557 subjects into two groups, one numbering 177 and the other 380. “Significance” was judged by the well-known duality between confidence intervals and p-values. Thus, for any 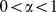 and null hypothesis that an unknown parameter 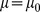, the p-value p = 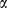 if, and only if, a 100(1-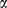)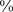 confidence interval for does not include 0. These being said, pooled t was done for 100 bootstrap samples, with 10,000 permutations done for each bootstrap sample, with bootstrapping done as described in Section 6. Results were about the same with B-F-W t as with pooled t. The Wilcoxon test is itself a permutation test. It was computed for each of the 100 bootstrap samples. Without giving exact p-values, we note that they agreed, at least approximately, with the normal approximation to B-F-W t on the original data. With cutoff of p = 0.05, the only features for which the fraction of rejections of the null hypothesis was not at least 99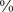 were Total Cholesterol and AGE.

### Predicting Cluster Membership by SNPs; Main Effects are not Enough, but Main Effects Together with Interactions may be

Three different sets of features were used for predicting cluster membership by each of the five algorithms introduced in the previous section. They were AGE + BMI alone, AGE+BMI+SNPs (main effects only), and AGE+BMI+SNPs (including their interactions chosen as cited); these are termed, respectively, Feature Set 1, Feature Set 2, Feature Set 3. Tables 7, 8, and 9 give cross-validated results for sensitivity and specificity for our best classifier, SVM, for various ratios of misclassification costs, as well as overall misclassification costs, for each of the three sets of features. We omit results by other four algorithms. The paired t-test averaged over the 10 folds in cross-validation with ratio of misclassification costs 2.2∶1 gave these values for comparison: Feature Set 1 versus Features Set 2, t = 0.939; Feature Set 2 versus Feature Set 3, t = 1.22; Feature Set 1 versus Feature Set 3, t = 2.38. Thus, there may be significantly good classification achieved by adding relevant SNPs and interaction terms to Feature Set 1. Graphical results not presented here demonstrate further that even when AGE and BMI are combined, they offer inadequate accuracy.

**Table 7:** SVM 10 fold cross-validation: AGE and BMI.

	Age+BMI			
Loss	Sensitivity	Specificity	Overall	Miscost
1.8∶1	0.432	0.743	0.638	242.8
1.9∶1	0.468	0.730	0.640	245.2
2.0∶1	0.552	0.682	0.633	242.9
2.1∶1	0.564	0.647	0.615	257.2
2.2∶1	0.589	0.613	0.6	266.4
2.3∶1	0.627	0.570	0.583	273.0

**Table 8:** SVM 10 fold cross-validation: Age, BMI and SNPs.

	Age+BMI+SNPs			
Loss	Sensitivity	Specificity	Overall	Miscost
1.8∶1	0.511	0.711	0.644	230.9
1.9∶1	0.526	0.692	0.635	240.2
2.0∶1	0.557	0.687	0.642	239.8
2.1∶1	0.569	0.667	0.633	249.2
2.2∶1	0.558	0.661	0.627	261.4
2.3∶1	0.561	0.633	0.608	276.7

**Table 9:** SVM 10 fold cross-validation: Age, BMI, SNPs and Interaction terms.

	Age+BMI+SNPs+Interaction terms			
Loss	Sensitivity	Specificity	Overall	Miscost
1.8∶1	0.504	0.704	0.638	235.3
1.9∶1	0.520	0.704	0.642	238.1
2.0∶1	0.526	0.697	0.640	246.0
2.1∶1	0.544	0.688	0.640	250.6
2.2∶1	0.580	0.682	0.646	247.3
2.3∶1	0.582	0.666	0.635	258.2

We did a standard chi-square test of the null hypothesis that classification is independent of cluster membership. The classifier chosen was the SVM with ratio of misclassification costs 2.1∶1. With this choice, 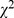 = 21.2. Of course, this is an example of a “maximally selected chi-square statistic,” and our cutoff is not linear in any test of features, as would be required for application of the argument of [63]. However, comparison of 21.2 with Table 2 of [63] suggests that the success of our classification does not owe to chance.

We asked whether adding SNPs and interactions to AGE and BMI might help predict cluster membership significantly. If we assume that SNPs do not improve the accuracy of classification, then classification would not change significantly by randomly permuting SNPs across people. We performed 1000 permutations and measured the area under the ROC curve of sensitivity versus 1-specificity for each permuted data set. We had the unpermuted data for comparison. For each permutation, we performed the permutation test for SNPs and interactions based on interactions computed for permuted SNPs. Without belaboring details, we report here that achieved significance (p-values) for improvements over Feature Set 1 by Feature Set 2 was 0.223, but that p-value was 0.028 when compared with Feature Set 3.

## Discussion

We have argued that prediction of cluster membership on the basis of SNPs and candidate genes and other features (that did not figure in the clustering) is better than could be expected by chance. However, our best algorithm, a support vector machine that includes interactions in its feature set, is not sufficiently accurate for routine clinical application. Instead, to the extent that genotype, other features, and their synergistic effects predict IR, it may be better to have data from a genome-wide association study (GWAS) than from candidate genes. This view is despite certain knowledge that any current GWAS necessarily entails beginning with not fewer than 500,000 features, the vast majority of them irrelevant, not to speak of the problem of describing “phenotype” (the dependent variable in prediction) accurately. Many previous analyses of GWAS (see [64], [65], [66], [67], for example) have focused primarily upon individual effects, no matter how minor any individual impact upon phenotype. This approach seems a vestige of thinking about Mendelian mechanisms of inheritance that do not port to this context of complex disease. Indeed, the argument for presenting our analyses is to demonstrate that statistics, broadly construed, can be brought to bear upon understanding prediction of complex human disease, and to show that despite the great care we took to define phenotype and to predict it as well as could be from information in “candidate genes, ” AGE, and BMI were simply not good enough in a family-based study like SAPPHIRe to lead to an algorithm for routine clinical application. Knowing proteins (in particular, genes that code for them) that figure in clinical presentation of a phenotype such as insulin resistance is not the same as knowing also what controls the expression or other aspects of those genes/proteins. Such control may depend on genes far removed on the genome from those that code for the particular protein. At present writing, at least so far as IR is concerned, these other genes tend to be unknown. Methods of causal inference and graphical models might be useful in this regard, but they are not the subject of this paper.
